# Application of three-dimensional visualization technology in phacoemulsification benefits the dry eye symptoms of patients after cataract surgery

**DOI:** 10.3389/fmed.2023.1247904

**Published:** 2024-01-16

**Authors:** Chen Wu, Qingzhong Chen, Guangbin Zhang

**Affiliations:** ^1^Xiamen Eye Center Affiliated to Xiamen University, Xiamen, China; ^2^Aier School of Ophthalmology, Central South University, Changsha, China

**Keywords:** three-dimensional, intraoperative light intensity, tear film stability, dry eye, cataract surgery three-dimensional, cataract surgery

## Abstract

**Purpose:**

To investigate the effects of the three-dimension visualization system on dry eye in patients after cataract phacoemulsification.

**Methods:**

Seventy-one patients (104 eyes) were enrolled in this study and assigned to the conventional microscopic group (CM group) or three-dimension group (3D group). Ocular Surface Disease Index, The Schirmer I test (SIt), lipid layer thickness (LLT), noninvasive tear breakup time (NIBUT) and other factors associated with dry eye were recorded before and 1 week and 1 month after surgery. The operation time and intraoperative light intensity (ILI) were also recorded.

**Results:**

The ILI in the 3D group was significantly lower than that in the CM group (33% vs. 60%, *p* < 0.01). There was an interaction (group and time) effect for first NIBUT (NIBUT-F), average NIBUT (NIBUT-Ave), tear meniscus height (TMH) and the score of eye redness (*P*_interaction_ < 0.05). The main effects of group on SIt, LLT, NIBUT-F, NIBUT-Ave and the score of eye redness were significant (*P*_group_ < 0.05). In the CM group, SIt, LLT, NIBUT-F, NIBUT-Ave, TMH were lower than those of the 3D group, the score of eye redness was higher than that of the 3D group at 1 week and 1 month after surgery (all *p* < 0.05). The changes in NIBUT-F and NIBUT-Ave between before surgery and 1 week after surgery showed negative correlations with ILI (*p* < 0.01).

**Conclusion:**

Compared with microscopic surgery, the 3D visualization system can provide better tear film stability for patients after cataract surgery.

## 1 Introduction

Although phacoemulsification cataract surgery (PCS) has improved the visual acuity of millions of individuals around the world over the past half century, there are potential complications, such as dry eye disease (DED) (1). Cataract surgery-associated dry eye means that surgery induced a disruption in tear film homeostasis (2). It is the main cause of early postoperative ametropia, reduction in visual quality, and reduction in patient satisfaction. There are several possible factors in modern PCS that can disturb the tear film (3). Light from the operating microscope are reported as one kind of factors that lead to postoperative dry eye (4). Phototoxic damage of the microscope to the ocular surface has been demonstrated in both animals and human studies (5). The NGENUITY three-dimensional (3D) visualization system (Alcon Laboratories) is a novel device that uses 3D glasses and a large, high-definition 3D monitor to present surgical images. The system offers advantages over a traditional microscope, including a high-resolution digital camera, which can provide better contrast and sharpness, and decreased illumination intensity. A previous study (6) reported that compared with traditional microsurgery, with the 3D visualization system, illumination intensity was decreased in patients and achieved the same effect as the traditional microscope during vitreoretinal surgery. Nariai et al. (7) found that a 3D surgical camera decreased illumination intensity by more than 50% during cataract surgery. However, the effects on dry eye of patients after cataract phacoemulsification from the 3D visualization system have not been evaluated before.

In this paper, we observed and compared the intraoperative light intensity (ILI) requirement between the 3D visualization system and traditional microscope; this study aimed to investigate the effects of different display devices on dry eye symptoms and tear film stability in patients after cataract phacoemulsification.

## 2 Methods

### 2.1 Patients

Seventy-one participants (104 eyes) with cataracts who underwent PCS at the Xiamen Eye Centre were enrolled in the study. The assignment of the participants was randomized according to the number table method. The inclusion criteria were patients over 18 years old with age-related cataract with normal ocular surface or mild DED (8); patients with a history of ocular trauma, corneal diseases, and corneal surgery were excluded. According to the visualization system, patients were assigned to the conventional microscopic group (CM group, 36 cases, 52 eyes) or three-dimensional group (3D group, 35 cases, 52 eyes). All patients in this study signed informed consent, the study adhered to the tenets of the Declaration of Helsinki and was approved by the Ethics Committee of Xiamen Eye Center affiliated of Xiamen University (XMYKZX-KY-2020-011).

### 2.2 Cataract surgery

All surgeries were performed by an experienced surgeon (GZ). At the beginning of each surgery, the surgeon adjusted the microscope light intensity to the lowest state required according to the needs of the operation and completed the operation under this state. To avoid bias as much as possible, the surgeon did not know the exact level of microscope light intensity until the surgery was finished. The real ILI during the surgery, operating time (OT), intraoperative phacoemulsification time (UST), cumulative dissipated energy (CDE), and fluid loss were recorded by the other researcher who was not involved in the surgery.

CM group: an OPMI-Lumera 700 operating microscope (Zeiss, Germany) was used throughout the procedure. After anesthesia and disinfection of the operative eye, a 2.2 mm clear corneal incision at the 11 point of the cornea limbal area and the corresponding lateral incision at the 3 point were performed under a binocular microscope. A 2% hydroxypropyl methylcellulose corneal protector (Bausch & Lomb) was used throughout the procedure to maintain surgical optical clarity and minimize the circulatory effects of irrigating the ocular surface after repeated drying. Annular capsulorhexis, water separation, and water stratification were performed. The lens phacoemulsification was performed with an Alcon Centurion phacoemulsification apparatus, and the residual cortex was aspirated by I/A. Intraocular lenses (Acrysof SN60WF, Alcon Laboratories) were implanted in the capsular bags. The hydroxypropyl methylcellulose was removed, the transparent corneal incision was hydrated to seal, tobramycin and dexamethasone were applied to the eyes, and the eyes were protected by a transparent eye mask.

3D group: the operative procedures were identical to those of the CM group, except a 3D visualization surgical system (Alcon Laboratories, United States) was connected to the same surgical microscope. The display was positioned approximately 1.6 m in front of the surgeon. The surgeon and assistants wore 3D polarized glasses to complete the whole operation, without looking through microscope eyepieces.

### 2.3 Subjective and objective index of dye eye

The Ocular Surface Disease Index (OSDI) questionnaire (9) contains 12 items that assess subjective symptoms of dry eye. In consideration of the significant impact of cataract surgery on vision, vision-related limitations were not included in the study. The score of each item depends on frequency, the final score is the sum of the scores of each item multiplied by 25 and divided by the number of items answered. A higher score means more severe symptoms.

The detection method of Schirmer I Test (SIt) was tear secretion test paper applied in the conjunctival capsule. Measured the length of the soaked paper after 5 min later.

Lipid Layer Thickness (LLT) was measured using a Lipiview Interferometer (TearScience, Morrisville, NC). The average value was recorded automatically.

Noninvasive Tear Breakup Time (NIBUT) (10), Tear Meniscus Height (TMH) (11), meibomian gland dropout score (MGDS) (12, 13), the score of eye redness (14) were measured automatically by a Keratograph 5 M (Oculus GmbH, Wetzlar, Germany) instrument. The degree of MGDS was scored on a 0–3 grade (13). The range of score of eye redness was from 0–4.

Sodium fluorescein was dripped into the conjunctival sac of the lower fornix. The phenomenon of corneal coloration was observed after blinking, the range of Corneal Fluorescein Staining Score (CFS) from 0 (lowest intensity) to 5 (highest intensity) (15).

### 2.4 Data record

Basic information of the two groups were recorded, included age, gender, and hardness grade of lens. Uncorrected visual acuity (UCVA), best corrected visual acuity (BCVA), intraocular pressure (IOP), corneal endothelial cell count (CECC), OSDI score, SIt, LLT, NIBUT, TMH, MGDS, CFS, and the score of eye redness were recorded before surgery and 1 week and 1 month after surgery.

Intraoperative and postoperative complications (intraoperative bleeding, posterior capsule rupture, iris root rupture, secondary glaucoma, corneal injury, endophthalmitis, chronic uveitis, etc.) were closely observed.

### 2.5 Statistical analysis

Statistical analysis was conducted using SPSS 26.0. The results are presented as the mean ± standard deviation and median. Normality was assessed using the Kolmogorov–Smirnov test. The chi-square test, rank sum test, two-factor repeated measures ANOVA were used to analyze the data.

Multiple comparison (LSD) test was used for pairwise comparisons within group, independent samples *t*-test was used for comparison between two groups. Pearson correlation tests were used for correlation analysis. *p* < 0.05 was considered statistically significant.

## 3 Results

### 3.1 Basic information

There were no significant differences in basic information between the two groups (all *p* > 0.05; Table 1). No significant differences were found in UCVA, BCVA, IOP and CECC between the two groups (all *p* > 0.05; Table 2).

**Table 1:** Comparison of intraoperative conditions between the two groups.

Group	Numbers of eyes	ILI(median)	UST (s,^−^*x* ± *s*)	Fluid loss (mL,^−^*x* ± *s*)	CDE (^−^*x* ± *s*)	OT (s,^−^*x* ± *s*)
3D group	52	33% (23.5, 48%)	28.58 ± 10.96	43.83 ± 9.47	4.68 ± 2.38	332.11 ± 35.35
CM group	52	60% (60, 60%)	25.44 ± 10.77	41.48 ± 10.68	4.84 ± 2.04	333.60 ± 55.68
t/Z		−6.95	1.48	1.19	−0.36	0.16
*P*		<0.01	0.14	0.24	0.72	0.87

ILI, intraoperative light intensity; UST, intraoperative phacoemulsification time; CDE, cumulative dissipated energy; OT, operating time; 3D, three-dimensional; CM, conventional microscopic. *P* < 0.05 was considered statistically significant.

**Table 2:** Subjective and objective index of dye eye between the two groups.

	OSDI score			SIt			LLT		
	Preop	1 w	1 m	Preop	1 w	1 m	Preop	1 w	1 m
3D group	10.19 ± 5.76	13.36 ± 6.46^bd^	10.46 ± 5.20	10.47 ± 2.10	8.83 ± 1.66^b^	9.33 ± 1.54^c^	77.71 ± 22.80	79.31 ± 19.37	80.33 ± 22.17
CM group	11.05 ± 5.75	13.50 ± 5.33^bd^	10.10 ± 4.94	9.96 ± 2.09	8.24 ± 1.18^ab^	8.72 ± 1.02^ac^	75.48 ± 22.08	69.96 ± 22.20^ab^	71.35 ± 21.85^a^
F	F_group_ = 0.05, F_time_ = 24.86, F_interaction_ = 0.78, *P*_group_ = 0.82, *P*_time_ < 0.001, *P*_interaction_ = 0.50, 0, 0.196, 0.008			F_group_ = 7.60, F_time_ = 31.89, F_interaction_ = 0.03, *P*_group_ < 0.01, *P*_time_ < 0.01, *P*_interaction_ = 0.96, 0.069, 0.238, 0			F_group_ = 4.83, F_time_ = 0.28, F_interaction_ = 1.34, *P*_group_ = 0.03, *P*_time_ = 0.75, *P*_interaction_ = 0.27, 0.045, 0.003, 0.013		
*P*									
Partial *η*2									
	NIBUT-F			NIBUT-Ave			TMH		
	Preop	1 w	1 m	Preop	1 w	1 m	Preop	1 w	1 m
3D group	9.55 ± 2.28	8.98 ± 3.22	9.49 ± 4.44	11.50 ± 2.39	10.26 ± 3.06	11.39 ± 4.24	0.16 ± 0.04	0.15 ± 0.03	0.16 ± 0.03
CM group	9.16 ± 2.66	6.79 ± 3.28^abd^	7.38 ± 3.93^ac^	11.35 ± 2.56	7.85 ± 3.25^abd^	9.76 ± 3.56^ac^	0.16 ± 0.04	0.14 ± 0.03^ab^	0.14 ± 0.03^ac^
F	F_group_ = 17.40, F_time_ = 11.11, F_interaction_ = 5.73, *P*_group_ < 0.01, *P*_time_ < 0.01, *P*_interaction_ < 0.01, 0.146, 0.198,0.053			F_group_ = 12.32, F_time_ = 15.10, F_interaction_ = 5.35, *P*_group_ < 0.01, *P*_time_ < 0.01, *P*_interaction_ < 0.01, 0.108, 0.129, 0.050			F_group_ = 1.78, F_time_ = 7.43, F_interaction_ = 4.01, *P*_group_ = 0.19, *P*_time_ < 0.01, *P*_interaction_ = 0.02, 0.017, 0.068, 0.038		
*P*									
Partial η2									
	MGDS			The score of eye redness			CFS		
	Preop	1 w	1 m	Preop	1 w	1 m	Preop	1 w	1 m
3D group	0.87 ± 0.34	0.90 ± 0.36	0.88 ± 0.43	1.31 ± 0.22	1.25 ± 0.19^d^	1.17 ± 0.14^c^	1.08 ± 0.81	0.94 ± 1.27	0.81 ± 1.37
CM group	0.81 ± 0.49	0.85 ± 0.46	0.83 ± 0.47	1.26 ± 0.16	1.37 ± 0.21^abd^	1.25 ± 0.17^a^	0.87 ± 0.84	1.19 ± 1.51	0.96 ± 1.45
F	F_group_ = 0.60, F_time_ = 0.66, F_interaction_ = 0, *P*_group_ = 0.44, *P*_time_ = 0.52, *P*_interaction_ = 1, 0.006, 0.006, 0			F_group_ = 4.94, F_time_ = 11.52, F_interaction_ = 7.25, *P*_group_ = 0.03, *P*_time_ < 0.01, *P*_interaction_ < 0.01, 0.046, 0.101, 0.066			F_group_ = 0.23, F_time_ = 0.54, F_interaction_ = 0.96, *P*_group_ = 0.63, *P*_time_ = 0.59, *P*_interaction_ = 0.39, 0.002, 0.005, 0.009		
*P*									
Partial η2									

OSDI, ocular surface disease index; SIt, schirmer I test; LLT, lipid layer thickness; NIBUT-F, first noninvasive tear breakup time; NIBUT-Ave, average noninvasive tear breakup time; TMH, tear meniscus height; MGDS, meibomian gland dropout score; CFS, corneal fluorescein staining score; 3D, three-dimensional; CM, conventional microscopic. The data was analyzed by two-factor repeated measures ANOVA. Multiple comparison (LSD) test was used for pairwise comparisons within group, independent samples *t*-test was used for comparison between two groups. *P* < 0.05 was considered statistically significant. F_group_, P_group_: the main effects of group; F_time_, P_time_: the main effects of time; F_interaction_, P_interaction_: interaction effect (group and time). Partial *η*2: the effect size, the larger the value indicates the larger the effect size.

a *p* < 0.05 the CM group compared with the 3D group at the same time point.

b *p* < 0.05 1 week postoperatively compared with preoperatively in the same group.

c *p* < 0.05 1 month postoperatively compared with preoperatively in the same group.

d *p* < 0.05 1 week postoperatively compared with one month postoperatively in the same group.

### 3.2 ILI and surgery time

The median ILI of the 3D group was 33% (23.5, 48%), significantly lower than that of the CM group 60% (60, 60%) (*p* < 0.01). There were no significant differences in OT, intraoperative UST, fluid loss, and CDE between the two groups (all *p* > 0.05; Table 1). No intraoperative or postoperative complications were found in each group.

### 3.3 Subjective and objective index of dye eye

The OSDI score and the other ocular surface function indexes, including SIt, LLT, NIBUT-F, NIBUT-Ave, TMH, MGDS, CFS and the score of eye redness showed no differences between the two groups preoperatively. There was an interaction (group and time) effect for NIBUT-F, NIBUT-Ave, TMH and the score of eye redness (*P*_interaction_ < 0.05), but there was no interaction effect (group and time) for the remaining factors (*P*_interaction_ > 0.05). The main effects of group on SIt, LLT, NIBUT-F, NIBUT-Ave, and the score of eye redness were significant (*P*_group_ < 0.05). The main effects of group on OSDI score, TMH, MGDS, and CFS were not significant (*P*_group_ > 0.05). The main effects of time on OSDI score, SIt, NIBUT-F, NIBUT-Ave, TMH, and the score of eye redness were significant (*P*_time_ < 0.05). The main effects of time on LLT, MGDS, and CFS were not significant (*P*_time_ > 0.05).

Compared with before surgery, OSDI score and the score of eye redness were increased in the CM group at 1 week after surgery, LLT in the CM group decreased at 1 week after surgery, SIt, NIBUT-F, NIBUT-Ave and TMH in the CM group decreased at two timepoints after surgery, the differences were statistically significant (all *p* < 0.05). OSDI scores in the 3D group increased significantly at 1 week after surgery(*p* = 0.02), SIt in the 3D group decreased significantly at two timepoints after surgery(*p* = 0.04), LLT, NIBUT-F, NIBUT-Ave, TMH in the 3D group at two timepoints after surgery showed no statistical differences as compared with before surgery (all *p* > 0.05). SIt, LLT, NIBUT-F, NIBUT-Ave, and TMH in the CM group were lower than those in the 3D group, the score of eye redness was higher than that in the 3D group at two timepoints after surgery, the differences were statistically significant (all *p* < 0.05). MGDS, CFS at any timepoints showed no significant differences between two groups (*p* > 0.05). Pairwise comparison of the three measurement time points in MGDS, CFS in each group showed no statistically significant differences (*p* > 0.05) (Table 2).

### 3.4 Correlation analysis

**Figure 1:**
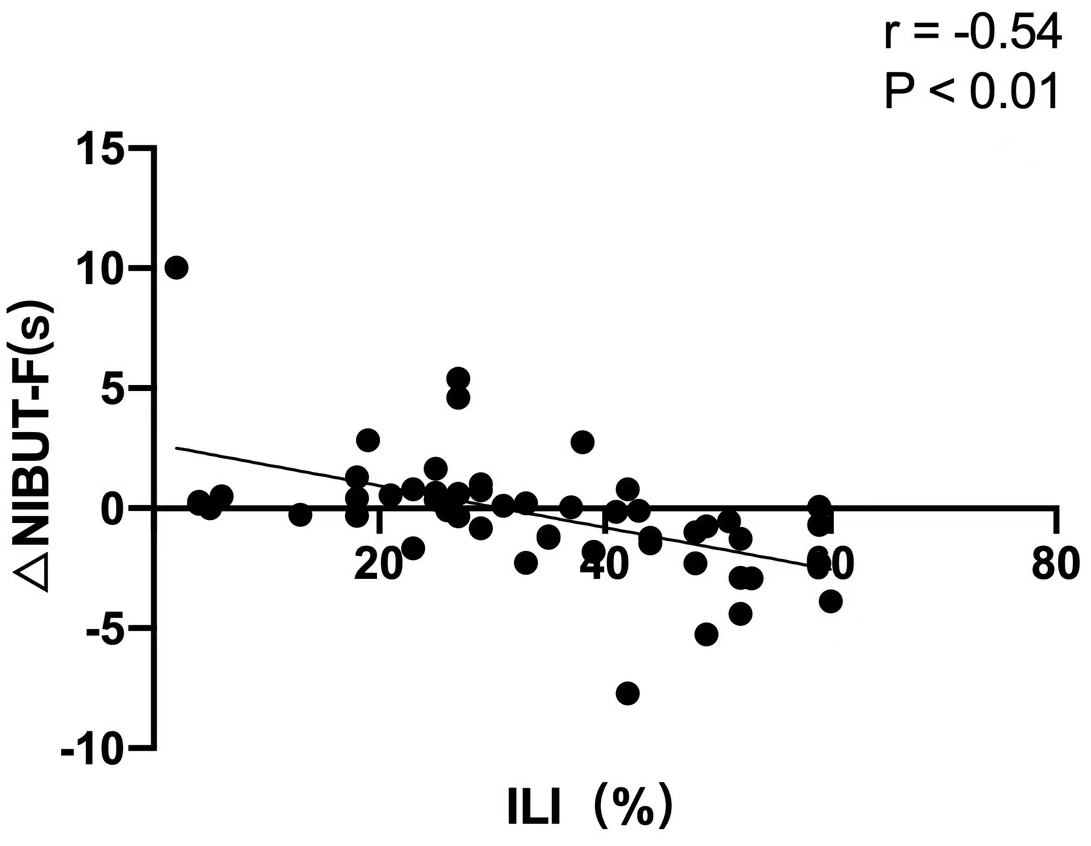
The changes in first noninvasive tear breakup time (NIBUT-F) between before surgery and 1 week after surgery showed a negative correlation with the intraoperative light intensity (ILI).

**Figure 2:**
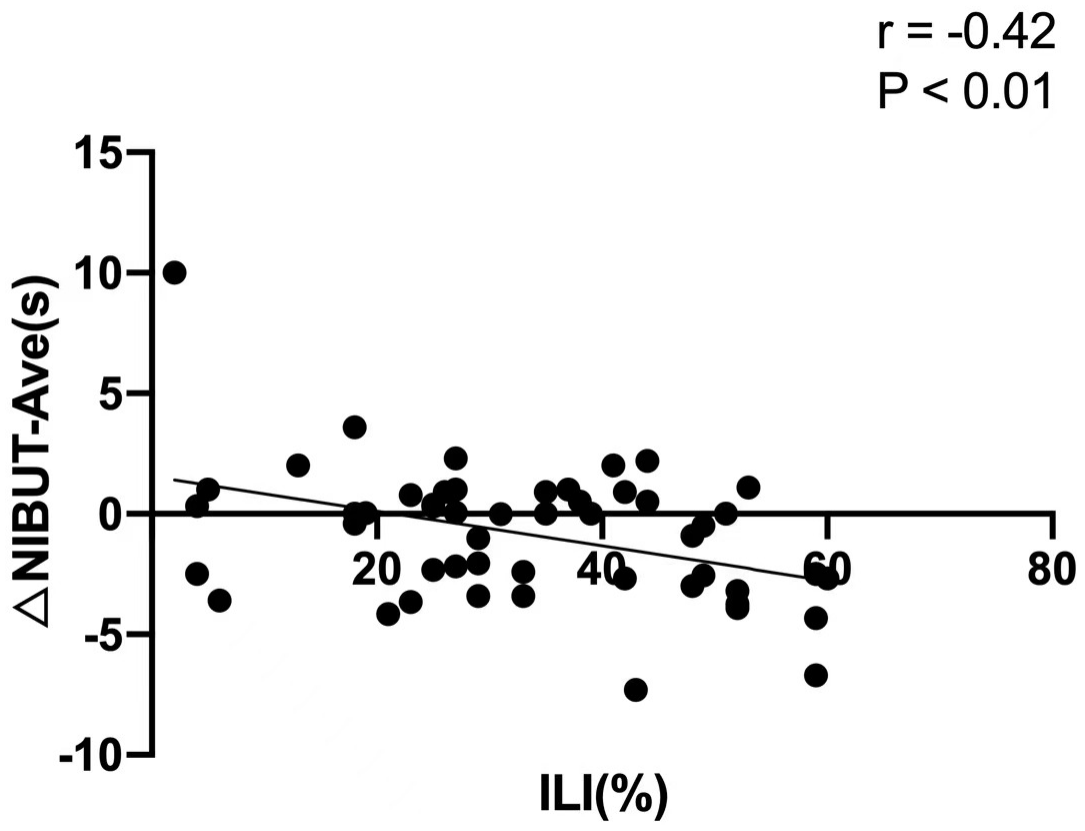
The changes in average noninvasive tear breakup time (NIBUT-Ave) between before surgery and 1 week after surgery showed a negative correlation with the intraoperative light intensity (ILI).

In 3D group, the changes in NIBUT-F between before surgery and 1 week after surgery (NIBUT-F_1w_ minus NIBUT-F_pre-op_) showed a negative correlation with ILI (*r* = −0.54, *p* < 0.01; Figure 1). The changes in NIBUT-Ave between before surgery and 1 week after surgery (NIBUT-Ave_1w_ minus NIBUT-Ave_pre-op_) showed a negative correlation with ILI (*r* = −0.42, *p* < 0.01; Figure 2).

## 4 Discussion

Cataract surgery can directly cause postoperative DED and exacerbate preexisting DED, leading to irritation, redness, foreign body sensation, and compromised visual outcomes (16, 17). A prospective study by Choi et al. (18) indicated that according to OSDI results, about one-third of patients experienced persistent dry eye symptoms after cataract surgery. Previous studies have demonstrated that reduced NIBUT and increased CFS after cataract surgery. Microscopic light exposure is an important cause of dry eye after cataract surgery (6). During eye surgery, the ocular surface is exposed to high light intensity at a close range, which makes the ocular surface vulnerable to thermal and photochemical damage. Although the light intensity of the microscope can be adjusted during surgery to avoid the influence of intraoperative light on ocular surface function, the reduction of the light intensity of a traditional microscope has a certain degree of limitation. Once the light intensity is lower than a certain degree, the vision of the surgeon will be affected, thus affecting the process of surgery. 3D systems use real-time digital signal processing on large displays to enhance visual quality and provide better depth of field and image resolution. Previous studies have shown that using the 3D visualization system can effectively reduce intraoperative illumination requirements. Adam et al. (19) found that conventional microscopic surgery required 40% of the maximum luminance probe illumination output, whereas the 3D system reduced the light level to 10% during vitreoretinal surgery. According to Eckardt and Paulo (7), performing vitreous surgery using a conventional microscope requires the surgeon to turn the light to 100% to achieve good intraoperative visibility, whereas using the 3D visualization system and turn the light to 60–80% can achieve the same effect. Theoretically, the 3D visualization system can reduce phototoxic damage of the patient’s ocular surface. Therefore, under the conditions of the same operating environment and procedure and the same medication before and after operation, we investigated the effects of the 3D visualization system and traditional microscopic surgery on dry eye in patients after cataract phacoemulsification.

Subjective indicators related to dry eye symptoms and objective indicators related to ocular surface function were measured. The OSDI is one of the most frequently used validated questionnaires regarding patient symptomatology in dry eye research. In our study, the OSDI score increased significantly in both groups at 1 week postoperatively and returned to baseline at 1 month. Choi et al. (18) has reported that OSDI score increased after cataract surgery under conventional microscope. After cataract surgery, the local eminence of the surgical incision may affect the distribution of tear film, leading to dry eye. Patients also difficult to determine whether pain or foreign body sensation is caused by dry eyes or by the surgical incision. All of these factors influenced the final OSDI score. Because the OSDI is based on patients’ subjective feelings and cannot fully reflect the ocular surface function of patients, we also used objective indicators to assess patients’ dry eye symptoms.

LLT and NIBUT are the principal indexes that reflect the stability of the tear film. The lipid layer is located in the outermost layer of the tear film. The function of lipid layer includes reducing tear evaporation, maintaining the stability of the tear film (20). Insufficient lipid layer thickness leads to tear film instability and increases the risk of dry eye. SIt and TMH reflect the secretory function of tears. The score of eye redness is an effective index to measure the symptoms of ocular surface irritation and the degree of conjunctival inflammation. Khanal et al. (21) reported statistically significant changes in tear production, evaporation, LLT, NIBUT, and corneal sensitivity after cataract surgery. Zamora et al. (22) found that the volume of tear secretion decreased in the short term after cataract surgery. Li et al. (23) also reported decreased SIT and TBUT during the postoperative period. In our study, SIt, LLT, NIBUT-F, NIBUT-Ave and TMH in the CM group significantly decreased, the score of eye redness in the CM group significantly increased after surgery, which is consistent with the results of previous studies (24). On the contrary, SIt decreased after surgery in the 3D group, but the degree of reduction was lower than that in the CM group. LLT, NIBUT-F, NIBUT-Ave, TMH and the score of eye redness in the 3D group showed no statistical differences compared with those before surgery. The result indicating that the 3D visualization system experienced little effect on tear secretion, tear film stability and the irritation symptoms of ocular surface after cataract surgery.

There was no statistical difference in OT between the two groups. The minimum ILI requirement in the 3D group was significantly lower than that in the CM group for safe surgery, and the surgery could be successfully completed with the same efficiency with only half of the ILI required by the traditional microscope during the operation under the 3D visualization system. According to previous studies (25–27), intraoperative microscopic light damage is the cause of tear film instability after cataract phacoemulsification, which may be caused by reactive oxygen species generated by microscope light irradiation, resulting in inactivation of the cornea and conjunctival epithelium, conjunctival squamosal hyperplasia, and decreased goblet cell density, thus lead to decreased tear film stability and imbalance of the ocular surface microenvironment. Even with the application of modern spectral filters and increased working distances, patients may stand light exposure beyond the safe range (28–30). The stronger ILI induced more severe irritation to the eyes. In this study, SIt, LLT, NIBUT-F, NIBUT-Ave, and TMH of the CM group were lower than those in the 3D group and the score of eye redness was higher than that of the 3D group at two timepoints after surgery. The results showed that the use of the 3D visualization system reduce ILI requirement could improve the signs of ocular surface in patients postoperatively.

To clarify the effect of ILI on DED, correlative analyses were conducted. At 1 week after surgery, negative correlations were found between the changes in NIBUT-F and NIBUT-Ave with ILI, which indicates that the stronger the ILI, the worse the stability of the tear film postoperatively. But the change in SIt and TMH was not correlated with ILI, which indicates compared with the secretory function of tears, ILI has a greater impact on tear film stability. Light from the surgical microscope had the following phototoxic effects on the ocular surface: increased levels of tear inflammatory cytokines (31), decreased expression of conjunctival mucin 5 AC (32, 33), and squamous metaplasia in conjunctival impression cytology (34). These changes were closely associated with tear film instability. The negative correlation of the change in NIBUT with ILI indicated that the use of the 3D visualization system to reduce ILI requirement plays a potential role in maintaining tear film stability and avoiding adverse effects on the ocular surface after cataract surgery.

Comparison between before and after surgery in MGDS in each group showed no statistically significant differences, which may be due to the fact that cataract surgery mainly worsened the functional expression of meibomian glands, and the operation time was short, with little influence on the structure of meibomian glands (35). There was also no significant difference in CFS before and after surgery in both groups. Oh et al. (36) reported that infiltration and fluorescence staining of the corneal epithelium were observed in most patients postoperatively, and the CFS scores increased immediately after surgery. Our results are different from the previous cases of CFS appearing rapidly on the first day after cataract surgery. The main reason may be that the ocular surface was exposed during the surgery, repeated irrigated ocular surface to maintain surgical optical clarity in previous cases (37). In our study, the use of 2% hydroxypropyl methylcellulose corneal protector avoided repeated drying followed by irrigation during the surgery.

There are some limitations to this study. First, although lubricating eye drops were not used, all patients were prescribed eye drops of antibiotics, corticosteroids, and nonsteroidal anti-inflammatory eye drops after surgery. Use of these agents may affect dry eye test values in the short term. To minimize the potential influence of the eye drops on the results, the participants enrolled in the study were treated with the same eye drop regimen and asked to cease ophthalmic medications 12 h before each follow-up measurement. Second, changes in corneal sensitivity after cataract surgery were not included in this study, and the effect of ILI on postoperative corneal sensitivity needs to be further studied.

In conclusion, compared with traditional surgery using the microscope, the 3D visualization system provides better tear film stability for patients after cataract surgery. The reason is that the use of the 3D visualization system can reduce the requirement for ILI and help to reduce phototoxic damage to the patient’s ocular surface.

## Data availability statement

The original contributions presented in the study are included in the article/supplementary material, further inquiries can be directed to the corresponding author.

## Ethics statement

The studies involving humans were approved by the Ethics Committee of Xiamen Eye Center Affiliated Xiamen University. The studies were conducted in accordance with the local legislation and institutional requirements. The participants provided their written informed consent to participate in this study.

## Author contributions

CW: brewing and designing experiments, conducting research, collecting data, analyzing and interpreting data, drafting articles, and statistical analysis. QC: data collection, statistical analysis, and critical review of the intellectual content of the article. GZ: design experiments, conduct studies, collect data, and critically review the intellectual content of the articles. All authors contributed to the article and approved the submitted version.
